# Long‐term preservation of kidney function with SGLT‐2 inhibitors versus comparator drugs in people with type 2 diabetes and chronic kidney disease

**DOI:** 10.1111/dom.16569

**Published:** 2025-07-02

**Authors:** Gian Paolo Fadini, Enrico Longato, Mario Luca Morieri, Fabio Broglio, Gianluca Aimaretti, Giuseppina T. Russo, Roberto Anichini, Mariangela Ghiani, Angelo Avogaro, Anna Solini, DARWIN‐Renal Study Investigators, Maria Pompea Antonia Baldassarre, Gloria Formoso, Agostino Consoli, Sara Morgante, Antonella Zugaro, Marco Giorgio Baroni, Francesco Andreozzi, Adriano Gatti, Stefano De Riu, Andrea Del Buono, Raffaella Aldigeri, Riccardo Bonadonna, Alessandra Dei Cas, Angela Vazzana, Monica Antonini, Valentina Moretti, Patrizia Li Volsi, Miranda Cesare, Giorgio Zanette, Silvia Carletti, Paola D'Angelo, Gaetano Leto, Frida Leonetti, Ernesto Maddaloni, Raffaella Buzzetti, Simona Frontoni, Maria Gisella Cavallo, Ilaria Barchetta, Susanna Morano, Tiziana Filardi, Umberto Capece, Andrea Giaccari, Antonio C. Bossi, Giancarla Meregalli, Fabrizio Querci, Alessia Gaglio, Veronica Resi, Emanuela Orsi, Stefano Fazion, Ivano G. Franzetti, Cesare Berra, Silvia Manfrini, Gabriella Garrapa, Giulio Lucarelli, Lara Riccialdelli, Elena Tortato, Marco Zavattaro, Gianluca Aimaretti, Franco Cavalot, Guglielmo Beccuti, Fabio Broglio, Bruno Fattor, Giuliana Cazzetta, Olga Lamacchia, Anna Rauseo, Salvatore De Cosmo, Rosella Cau, Mariangela Ghiani, Antonino Di Benedetto, Antonino Di Pino, Salvatore Piro, Francesco Purrello, Lucia Frittitta, Agostino Milluzzo, Giuseppina Russo, Anna Solini, Monia Garofolo, Giuseppe Penno, Stefano Del Prato, Roberto Anichini, Gian Paolo Fadini, Angelo Avogaro, Mauro Rigato, Agostino Paccagnella, Marco Strazzabosco, Massimo Cigolini, Enzo Bonora

**Affiliations:** ^1^ Department of Medicine University of Padova Padova Italy; ^2^ Veneto Institute of Molecular Medicine Padova Italy; ^3^ Department of Information Engineering University of Padova Padova Italy; ^4^ Division of Endocrinology, Diabetes and Metabolism, Department of Medical Sciences University of Turin Turin Italy; ^5^ Endocrinology, Department of Translational Medicine Università del Piemonte Orientale Novara Italy; ^6^ Department of Clinical and Experimental Medicine University of Messina Messina Italy; ^7^ Diabetic Unit and Diabetic Foot Unit San Jacopo Hospital Pistoia Pistoia Italy; ^8^ Diabetology Unit Azienda Sanitaria Locale 8 Cagliari Quartu S. Elena Cagliari Italy; ^9^ Department of Surgical, Medical, Molecular and Critical Area Pathology University of Pisa Pisa Italy

**Keywords:** gliflozin, observational, outcomes, renal, retrospective

## Abstract

**Aims:**

Chronic kidney disease (CKD) is a prevalent and serious complication of type 2 diabetes (T2D). This study aims to evaluate kidney outcomes in a real‐world cohort of patients with T2D and CKD who received SGLT2 inhibitors (SGLT2i) or other glucose‐lowering medications (GLM).

**Materials and Methods:**

This retrospective, multicentre study analysed data from patients aged 18–80 years with T2D and CKD, who initiated an SGLT2i or other GLM between 2015 and 2020. The primary outcome was the change in estimated glomerular filtration rate (eGFR) over time. Secondary outcomes included albuminuria changes and adverse kidney events. Propensity score matching was used to balance baseline characteristics between the two groups.

**Results:**

After matching (*n* = 2020/group), patients (100% T2D with CKD) had a mean age of 63 years, BMI 32 kg/m^2^, HbA1c 8.2%. New‐users of SGLT2i exhibited a slower decline in eGFR compared with new users of comparators (mean difference 1.43 mL/min/1.73 m^2^; *p* = 0.048). Albuminuria improved significantly more in the SGLT2i group, with a greater likelihood of category improvement (hazard ratio [HR] 1.17; *p* = 0.007). SGLT2i initiation was associated with a lower incidence of kidney outcomes, including a ≥40% eGFR reduction (HR 0.63; *p* = 0.004). When the comparison was restricted to SGLT2i versus GLP‐1RA (*n* = 1266/group), the eGFR slope was significantly better with SGLT2i (mean difference 0.62 mL/min/1.73 m^2^/year; *p* = 0.046).

**Conclusions:**

In this large, real‐world cohort, initiation of SGLT2i was associated with a significantly slower decline in kidney function and improved albuminuria compared with other diabetes drugs, including GLP‐1RA. These findings support SGLT2i as the most effective T2D treatment to slow CKD progression.

## 1 INTRODUCTION

Chronic kidney disease (CKD) is one of the most frequent and serious complications of type 2 diabetes (T2D), affecting approximately 30%–40% of individuals with T2D and accounting for a substantial proportion of excess cardiovascular morbidity, premature mortality and healthcare expenditures in this population.^1^, ^2^

Diabetic kidney disease is clinically heterogeneous, and the classical phenotype characterized by albuminuria and progressive loss of estimated glomerular filtration rate (eGFR) coexists with non‐albuminuric forms, in which eGFR decline occurs in the absence of mild or overt proteinuria. This phenotypic variability reflects differences in underlying pathophysiology,^3^ may influence prognosis, and contributes to the complexity of managing CKD in people with T2D.^4^, ^5^, ^6^

Several therapeutic strategies have been shown to delay CKD progression in T2D.^7^ In addition to renin–angiotensin system (RAS) blockers,^8^ newer pharmacological agents have emerged with kidney‐protective effects. Sodium–glucose cotransporter 2 inhibitors (SGLT2i) significantly reduce the risk of CKD progression and cardiovascular events in patients with T2D and established CKD or high cardiovascular risk.^9^, ^10^ Non‐steroidal mineralocorticoid receptor antagonists (nsMRA), such as finerenone, further reduce albuminuria and adverse kidney and cardiovascular outcomes in patients with albuminuric CKD already receiving optimized background therapy.^11^, ^12^ More recently, glucagon‐like peptide‐1 receptor agonists (GLP‐1RA) have shown potential kidney protective effects, with the FLOW trial demonstrating a reduced risk of major kidney outcomes in T2D patients with CKD treated with semaglutide.^13^

Although randomized controlled trials have firmly established the efficacy of these therapies in selected populations,^14^ their translation into routine clinical practice remains suboptimal. Furthermore, the generalizability of trial findings to real‐world populations—who are often older, more comorbid, and more heterogeneous than trial participants—remains uncertain. Real‐world evidence is therefore essential to complement trial data, assess effectiveness across broader patient groups, and identify residual gaps in care.^15^ Moreover, given the lack of head‐to‐head comparisons in randomized clinical trials, real‐world studies offer a unique opportunity to provide initial insights into identifying the most effective treatments for patients in the context of an expanding range of beneficial therapeutic options. Yet, comparative observations of how different drugs perform in terms of nephroprotection in a real‐life context are scarce.

The present study addresses these knowledge gaps. Specifically, it aims to characterize kidney outcomes in new‐users of SGLT2i versus new‐users of other glucose‐lowering medications (GLM) in a large, contemporary cohort of patients with T2D and CKD in a real‐world clinical setting.

## 2 MATERIALS AND METHODS

### 2.1 Study design

DARWIN‐Renal was a retrospective, multicentre study promoted and conducted by the Italian Diabetes Society. The primary aim of the study was to compare kidney outcomes of patients who initiated dapagliflozin in the real world, but the objectives were then extended to consider SGLT2i as a class. Study design and the main results have been published before.^16^, ^17^ The protocol agrees with the declaration of Helsinki and was approved by the Ethics Committee of all participating centers. According to the national regulation on retrospective studies using anonymized data, patient's informed consent was waived. We followed the STROBE checklist for matched cohorts. Here, we present a predefined analysis comparing patients with T2D and CKD initiating any SGLT2i or comparator GLM.

### 2.2 Cohorts and exposure

The data collection period was from 1 January 2015 to 30 September 2020. We included patients aged 18–80 years, with T2D and CKD. CKD was defined as a reduction of eGFR below 60 mL/min/1.73 m^2^ or a raise in UACR above 30 mg/g. We selected two groups of patients: (i) new‐users of SGLT2i (dapagliflozin, empagliflozin, canagliflozin, ertugliflozin) and (ii) new‐users of any other GLM, excluding SGLT2i and insulin. The index date was the day when patients were prescribed for the first time one of these index drugs. Patients could be included if they had at least one eGFR value post index date. Exclusion criteria were: other forms of diabetes; prior therapy with SGLT2i; concomitant initiation of insulin; CKD stage V (eGFR < 15 mL/min/1.73 m^2^) or dialysis at baseline.

### 2.3 Data collection

Clinical data were extracted from the electronic chart at each of the 50 participating centres, as described before.^16^, ^18^ Briefly, at the index date (within −90 days) and at each follow‐up time point, we recorded demographics, anthropometrics, blood pressure, laboratory data and presence or absence of chronic complications and ongoing medications. Pre‐index‐date eGFR values were recorded in the database to calculate the baseline eGFR slope. The database did not contain structured information on adverse events occurring during treatment.

### 2.4 Endpoints and follow‐up

The primary endpoint was the change in eGFR from baseline through the follow‐up period. eGFR was calculated according to the CKD‐EPI equation.^19^ Secondary outcomes included: eGFR slopes and changes in UACR. Using eGFR and UACR values, we explored a few categorical outcomes: worsening in CKD class (stage I eGFR ≥ 90; stage II 60–90; stage IIIa 45–60; stage IIIb 30–45; stage VI 15–30; stage V < 15 mL/min/1.73 m^2^); loss of kidney function (defined as an eGFR reduction of 40% or greater relative to baseline value); doubling of serum creatinine (equal to a reduction of eGFR of 57% or greater relative to baseline value); improving albuminuria class (class 1: 0–30 mg/g; class II: 31–300 mg/g; class 3: 300 mg/g). ESKD (end‐stage kidney disease, defined as a confirmed eGFR <15 mL/min/1.73 m^2^ in at least two occasions at least 90 days apart); initiation of dialysis. A composite kidney endpoint included 40% or more reduction in eGFR, ESKD or dialysis. An extended composite endpoint also included new‐onset macroalbuminuria (occurrence of an UACR value of 300 mg/g or greater in participants with a baseline value below such threshold). We also recorded intermediate outcomes (HbA1c, body weight, systolic and diastolic blood pressure). Patients were observed for these endpoints until the last available visit, meaning they were censored only at last observation, as data on competing events (e.g., cardiovascular events) were not available. For those with a last observation occurring before follow‐up closure, we had no information on the reasons for censoring (i.e., death or lost to follow‐up).

### 2.5 Statistical analysis

Continuous variables are expressed as mean and standard deviation, while categorical variables are reported as numbers and percentages. Comparisons between two groups were performed using the Wilcoxon–Mann–Whitney test for continuous variables or the chi square test for categorical variables. The change over time in continuous variables was compared between the two groups using the mixed model for repeated measures (MMRM): eGFR was used as the dependent variable; treatment group (SGLT2i vs. comparators), time, the group × time interaction, and baseline eGFR were entered as fixed effects. The variance structure was heterogeneous compound symmetry. The output of the MMRM was the marginal means in each group and the mean difference between groups, and their standard errors. Categorical outcomes were compared using the Cox proportional hazards model, reporting hazard ratios (HR) and 95% confidence intervals (CI).

Given the expected differences between the baseline characteristics of the two groups, we performed propensity score (PS) matching (PSM) to obtain comparable groups. PS were calculated from a logistic regression with covariates listed in Table 1, chosen using the modified disjunctive cause criterion.^20^ Patients from the two groups were matched 1:1 with a calliper of 0.01 pooled SD using the nearest neighbour method without replacement. The balance between groups before and after PSM was confirmed by standardized mean differences (SMD) <0.1. As PSM requires complete cases, we applied multiple imputation by chained equations (MICE) to obtain 10 imputed datasets. The analyses were run on each imputed dataset and results were pooled. Outcome analyses in matched cohorts were performed with an unpaired approach, because the balance of clinical characteristics was reached only between groups and not within matched pairs. This approach yields results that do not deviate significantly from those obtained with a paired analysis.^16^

**TABLE 1:** Characteristics of the study population.

	Before PSM			After PSM		
	SGLT2i	Comparators	SMD	SGLT2i	Comparators	SMD
Number	3031	7789		2027	2027	
Demographics						
Sex male, %	2014 (66.4)	5062 (65.0)	0.03	1365 (67.3)	1367 (67.4)	<0.01
Age, years	63.0 (8.7)	66.1 (7.7)	0.39	63.4 (8.5)	63.4 (9.1)	<0.01
Diabetes duration, years	13.2 (9.0)	11.9 (8.3)	0.16	11.8 (8.7)	11.7 (8.7)	0.01
Anthropometrics						
Weight, kg	91.5 (18.7)	85.1 (17.6)	0.36	89.6 (17.8)	89.2 (19.0)	0.02
Height, cm	167.6 (9.5)	166.7 (9.5)	0.10	167.7 (9.6)	167.7 (9.5)	<0.01
Body mass index, kg/m^2^	32.5 (6.0)	30.6 (5.8)	0.33	31.9 (5.7)	31.7 (6.1)	0.03
Waist circumference, cm	111.3 (13.8)	107.3 (13.4)	0.30	110.4 (14.0)	109.9 (14.0)	0.04
Risk factors and laboratory						
Systolic blood pressure, mm Hg	140.1 (19.7)	137.9 (19.4)	0.11	139.4 (19.6)	139.4 (18.8)	<0.01
Diastolic blood pressure, mm Hg	79.2 (10.4)	78.0 (10.3)	0.11	79.3 (10.5)	79.3 (10.5)	<0.01
Fasting plasma glucose, mg/dL	177.6 (61.1)	157.1 (49.2)	0.39	169.9 (55.3)	169.9 (54.5)	<0.01
HbA1c, %	8.5 (1.5)	7.7 (1.3)	0.57	8.2 (1.4)	8.2 (1.4)	<0.01
Total cholesterol, mg/dL	172.6 (44.0)	168.9 (41.7)	0.09	171.6 (43.0)	171.1 (43.1)	0.01
HDL cholesterol, mg/dL	44.6 (12.8)	45.8 (13.9)	0.09	45.1 (12.7)	45.3 (13.4)	0.01
LDL cholesterol, mg/dL	93.3 (36.0)	90.6 (34.7)	0.08	92.9 (35.2)	92.0 (35.5)	0.02
Triglycerides, mg/dL	179.9 (119.2)	169.9 (114.3)	0.09	173.3 (111.9)	174.5 (112.0)	0.01
eGFR (mL/min/1.73 m^2^)	78.0 (21.3)	61.7 (23.9)	0.70	76.1 (21.0)	76.7 (24.0)	0.02
UACR, mg/g	250.9 (627.9)	217.5 (750.9)	0.05	234.3 (624.1)	234.9 (513.6)	<0.01
eGFR slope (mL/min/1.73 m^2^/year)	−1.5 (14.1)	−2.8 (18.6)	0.08	−1.9 (15.7)	−2.0 (10.7)	<0.01
Complications						
eGFR >15 and <60 mL/min/1.73 m^2^	884 (29.2)	4658 (59.8)	0.64	656 (32.4)	633 (31.2)	0.02
Pathologic albuminuria, %	2364 (78.0)	4208 (54.0)	0.50	1526 (75.3)	1546 (76.3)	0.02
Diabetic retinopathy, %	832 (27.4)	1456 (18.7)	0.22	424 (20.9)	429 (21.2)	<0.01
Diabetic macular edema, %	139 (4.6)	215 (2.8)	0.10	81 (4.0)	73 (3.6)	0.02
Stroke/TIA, %	55 (1.8)	182 (2.3)	0.04	30 (1.5)	33 (1.6)	0.01
Carotid atherosclerosis, %	778 (25.7)	2157 (27.7)	0.05	490 (24.2)	511 (25.2)	0.02
Ischaemic heart disease, %	552 (18.2)	1183 (15.2)	0.08	337 (16.6)	343 (16.9)	<0.01
Left ventricular hypertrophy, %	328 (10.8)	865 (11.1)	<0.01	216 (10.7)	195 (9.6)	0.03
Heart failure, %	141 (4.7)	336 (4.3)	0.02	85 (4.2)	77 (3.8)	0.02
Any site revascularization, %	376 (12.4)	861 (11.1)	0.04	232 (11.4)	233 (11.5)	<0.01
Established CVD, %	654 (21.6)	1502 (19.3)	0.06	402 (19.8)	400 (19.7)	<0.01
Glucose‐lowering medications						
Metformin, %	2271 (74.9)	4892 (62.8)	0.26	1580 (77.9)	1600 (78.9)	0.02
Sulphonylurea/repaglinide, %	170 (5.6)	1578 (20.3)	0.40	166 (8.2)	182 (9.0)	0.03
DPP‐4 inhibitors, %	46 (1.5)	973 (12.5)	0.38	46 (2.3)	40 (2.0)	0.02
GLP‐1RA, %	61 (2.0)	229 (2.9)	0.06	57 (2.8)	59 (2.9)	<0.01
Pioglitazone, %	40 (1.3)	283 (3.6)	0.14	40 (2.0)	41 (2.0)	<0.01
Acarbose, %	18 (0.6)	69 (0.9)	0.03	13 (0.6)	10 (0.5)	0.02
Bolus insulin, %	1153 (38.0)	315 (4.0)	1.11	282 (13.9)	271 (13.4)	0.02
Basal insulin, %	1584 (52.3)	1612 (20.7)	0.73	668 (33.0)	660 (32.6)	<0.01
Other medications						
Statins, %	1943 (64.1)	4703 (60.4)	0.08	1251 (61.7)	1259 (62.1)	<0.01
Anti‐platelet agents, %	1503 (49.6)	3660 (47.0)	0.05	938 (46.3)	967 (47.7)	0.03
RAS blockers, %	2191 (72.3)	5373 (69.0)	0.07	1428 (70.4)	1417 (69.9)	0.01
Beta blockers, %	1091 (36.0)	2683 (34.4)	0.03	691 (34.1)	684 (33.7)	<0.01
Calcium channel inhibitors, %	882 (29.1)	2342 (30.1)	0.02	604 (29.8)	580 (28.6)	0.03
Diuretics, %	1140 (37.6)	3348 (43.0)	0.11	769 (37.9)	747 (36.9)	0.02
Anticoagulants, %	107 (3.5)	443 (5.7)	0.10	81 (4.0)	72 (3.6)	0.02

*Note*: Data are shown before and after propensity score matching (PSM). The standardized mean difference (SMD) is reported to evaluate the balance between groups.

Abbreviations: CVD, cardiovascular disease; eGFR, estimated glomerular filtration rate; GLP‐1RA, glucagon‐like peptide‐1 receptor agonists; HDL, high‐density lipoprotein; LDL, low‐density lipoprotein; RAS, renin angiotensin system; SGLT2i, sodium–glucose cotransporter 2 inhibitors; TIA, transient ischemic attack; UACR, urinary albumin creatinine ratio.

The primary analysis was performed in the intention‐to‐treat (ITT) population, composed of all new users of index drugs who had at least one eGFR value post‐index date, censored at event occurrence (for time‐to‐event analyses) or last observation. We performed a sensitivity analysis on the on‐treatment (OT) population, censoring patients at the time of index drug discontinuation, the event, or last observation, whichever occurred first. Data on pharmacy refill rates and adherence were not available, and drug discontinuation occurred when the index drug was no longer prescribed at a follow‐up visit.

The conventional statistical significance threshold of 0.05 was used, without controlling for type I error inflation due to multiple endpoint testing. There was no hierarchy of statistical testing, except that secondary endpoints could be analysed only after showing a significant difference for the primary endpoint. The analyses were run in R 4.2.2, using the MatchIt, mice, glmmTMB, stats, and survival packages.

## 3 RESULTS

### 3.1 Patient disposition and characteristics

The dataset initially included 48 593 new users of glucose‐lowering medications. After excluding those without data to define the primary outcome and those initiating insulin, 42 388 patients remained. Of them, 10 820 had CKD, divided into 3031 initiators of SGLT2i and 7789 initiators of comparator drugs.

Before PSM, SGLT2i initiators were younger, with a longer disease duration, higher BMI and HbA1c, worse lipid profile, more prevalent retinopathy, and more concomitant use of insulin, but higher baseline eGFR and lower pre‐index date eGFR slope (Table 1).

After PSM, the mean sample size across the 10 imputed datasets was 2020 patients/group, which were very well balanced for all clinical features (all SMD < 0.1). In the first imputed dataset (Table 1), patients had a mean age of 63 years, a diabetes duration of about 12 years, BMI around 32 kg/m^2^, and baseline HbA1c of 8.2%. All participants had CKD defined by eGFR and/or UACR: baseline eGFR was 76 mL/min/1.73 m^2^, 31% had an eGFR <60 mL/min/1.73 m^2^, and 75% had an UACR >30 mg/g (6% had both reduced eGFR and elevated UACR). Forty‐two percent had at least one macrovascular complication. Concomitant GLM were primarily metformin (78%) and insulin (14% bolus, 33% basal). Seventy‐percent of patients were on RAS blockers.

In the SGLT2i group, patients initiated dapagliflozin (51%), empagliflozin (40%) or canagliflozin (9%). In the comparator group, patients initiated GLP‐1RA (38%), DPP‐4 inhibitors (38%), sulphonylureas (12%), metformin (5%) or pioglitazone (6%).

### 3.2 Change in eGFR

**FIGURE 1:**
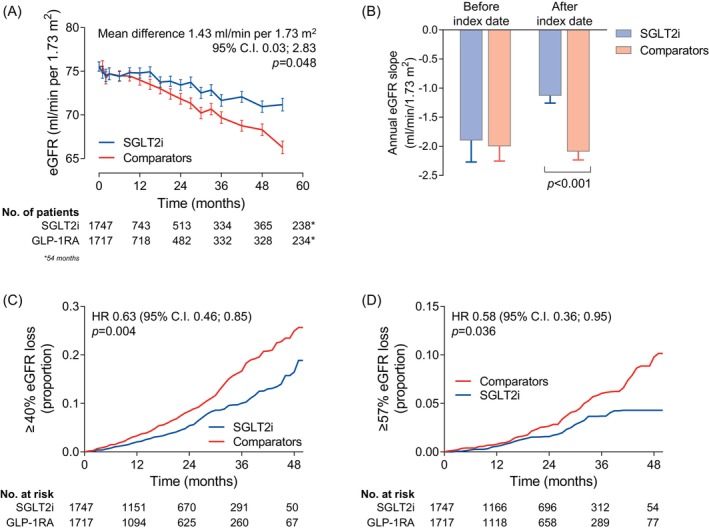
Estimated glomerular filtration rate (eGFR)‐based outcomes. (A) Change in eGFR over time in the two groups. The table shows number of patients contributing to the analysis at each timepoint. (B) eGFR slopes before and after index date. (C, D) Event rate curves for the ≥40% (C) or ≥57% loss of kidney function. The table shows the number of patients at risk at each timepoint. CI, confidence interval; GLP‐1RA, glucagon‐like peptide‐1 receptor agonists; HR, hazard ratio; SGLT2i, sodium–glucose cotransporter 2 inhibitors.

The median (interquartile range [IQR]) follow‐up time was 1.6 (0.7–2.9) years, during which there were (median and IQR) 6^3^, ^4^, ^5^, ^6^, ^7^, ^8^, ^9^, ^10^, ^11^ eGFR measurements per patient. From a baseline value of about 76 mL/min/1.73 m^2^, the eGFR declined significantly less in the SGLT2i than in the comparator group (Figure 1A). During observation, the eGFR remained significantly higher in the SGLT2i group than in the comparator group (mean difference 1.43 mL/min/1.73 m^2^; 95% CI 0.03–2.83; *p* = 0.048).

The eGFR curves before index date, built from a median (IQR) of 7^4^, ^5^, ^6^, ^7^, ^8^, ^9^, ^10^, ^11^, ^12^, ^13^ eGFR values per patient, were superimposable in the two groups (Figure S1). After the index date, the eGFR loss over time was significantly slower in the SGLT2i group, while it continued to decline linearly in the comparator group.

The annualized eGFR slope was very similar between groups (−2.0 mL/min/1.73 m^2^) before the index date, but diverged significantly after the index date: it remained −2.1 mL/min/1.73 m^2^ in the comparator group while it improved to −1.1 mL/min/1.73 m^2^ in the SGLT2i group (Figure 1B). The difference in the annualized eGFR slope after the index date was 0.96 (95% CI 0.58–1.35) mL/min/1.73 m^2^/year.

The categorical outcomes based on eGFR were in favour of the SGLT2i group. The HR of losing 40% or more eGFR was 0.63 (95% CI 0.46–0.85; *p* = 0.004; Figure 1C) and that of ≥57% eGFR loss was 0.58 (95% CI 0.36–0.95; *p* = 0.036; Figure 1D). The composite kidney outcome occurred less frequently in the SGLT2i versus the comparator group (HR 0.62; 95% CI 0.46–0.84; *p* = 0.003), but it was largely driven by the lower occurrence of ≥40% loss of kidney function.

The change in eGFR was re‐analysed separately in participants with normal UACR (<30 mg/g), who were included for having a reduced eGFR or raised UACR (≥30 mg/g). In the non‐albuminuric group, eGFR remained higher in the SGLT2i versus comparator groups by 3.8 mL/min/1.96 m^2^ (95% CI 0.6–7.1; *p* = 0.019). In the albuminuric group (94% with preserved eGFR) eGFR remained significantly higher by 1.4 mL/min/1.73 m2 (95% CI 0.1–2.8; *p* = 0.047).

### 3.3 Change in albuminuria

**FIGURE 2:**
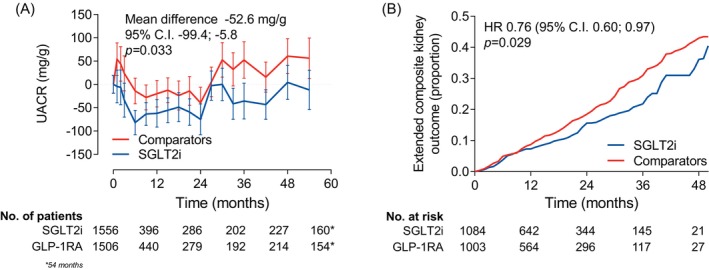
Albuminuria‐based outcomes. (A) Change in the urinary albumin excretion rate (UACR) over time. The table shows number of patients contributing to the analysis at each timepoint. (B) Event rate curves for the extended composite outcome including ≥40% loss of kidney function, new‐onset macroalbuminuria, end‐stage kidney disease, or dialysis. The table shows the number of patients at risk at each timepoint. CI, confidence interval; GLP‐1RA, glucagon‐like peptide‐1 receptor agonists; HR, hazard ratio; SGLT2i, sodium–glucose cotransporter 2 inhibitors.

UACR declined significantly in the SGLT2i versus the comparator group within 6 months after index date and remained significantly lower for the entire observation period, with a mean difference of −52.6 mg/g (95% CI −99.4 to −5.8; *p* = 0.033; Figure 2A). More patients in the SGLT2i group experienced UACR category improvement compared with those in the comparator group, with an HR of 1.17 (95% CI 1.05–1.31; *p* = 0.007). The result remained unchanged when a confirmatory UACR value was requested to meet this outcome (HR 1.19; 95% CI 1.05–1.35; *p* = 0.009).

The extended composite kidney outcome (including new‐onset macroalbuminuria) was less frequent in the SGLT2i group (HR 0.76; 95% CI 0.60–0.97; *p* = 0.029; Figure 2B).

### 3.4 Intermediate outcomes

HbA1c declined more in the comparator than in the SGLT2i group, with a mean difference of 0.16%. On the other side, systolic blood pressure declined more in the SGLT2i than in the comparator group by 1.7 mm Hg. No significant differences were observed for the change in diastolic blood pressure and body weight (Table S1).

### 3.5 Findings in the on‐treatment dataset

Results were largely confirmed in the OT dataset, wherein patients were censored at the time of discontinuation of index drugs. During a median (IQR) observation of 1.6 (0.7–3.0) years, eGFR declined less in the SGLT2i group with a mean difference of 1.51 (95% CI 0.11–2.91) mL/min/1.73 m^2^ than in the comparator group (*p* = 0.025). eGFR‐based categorical outcomes confirmed findings in the ITT dataset (except for the change in CKD class; Table 2).

**TABLE 2:** Major study outcomes in the intention‐to‐treat and on‐treatment datasets.

	Intention‐to‐treat		On treatment	
	HR (95% CI)	*p*‐Value	HR (95% CI)	*p*‐Value
Composite kidney outcome	0.62 (0.46–0.84)	0.003	0.56 (0.38–0.84)	0.007
Change in CKD class	0.90 (0.81–0.99)	0.038	0.95 (0.85–1.06)	0.329
≥40% eGFR loss	0.63 (0.46–0.85)	0.004	0.57 (0.38–0.84)	0.008
≥57% eGFR loss	0.58 (0.36–0.95)	0.036	0.52 (0.28–0.95)	0.041
Albuminuria class improvement	1.17 (1.05–1.31)	0.007	1.17 (1.03–1.33)	0.015

*Note*: For the two datasets, the table reports hazard ratios (HR) with 95% confidence interval (CI) and the respective *p*‐values.

Abbreviations: CKD, chronic kidney disease, eGFR, estimated glomerular filtration rate.

UACR declined in the SGLT2i group and remained significantly lower than in the comparator group by −54.6 mg/g (95% CI −103.6 to −5.5; *p* = 0.038). UACR category improvement occurred more frequently in the SGLT2i group (Table 2).

### 3.6 Comparison of eGFR slope between new‐users of SGLT2i versus GLP‐1RA

The analysis, including MICE and PSM, was repeated comparing new‐users of SGLT2i with new users of GLP‐1RA. Across the 10 imputed datasets, 1226 patients/group were included. The first imputed dataset is shown in Table S2, illustrating that clinical characteristics were well balanced after PSM and were similar to those described for the main analysis comparing SGLT2i versus all comparators.

GLP‐1RA were distributed as follows: dulaglutide (51%), liraglutide (34%), exenatide (9%), semaglutide (4%), lixisenatide (2%).

Before the index date, the annual eGFR slope was −2.1 and −2.0 mL/min/1.73 m^2^ in the SGLT2i and in the GLP‐1RA group, respectively. After the index date, the annual eGFR slope improved to −1.0 and −1.6 mL/min/1.73 m^2^ in the SGLT2i and in the GLP‐1RA group, respectively (Figure S2). The mean difference in the annual eGFR slope was 0.62 (95% CI 0.02–1.22) mL/min/1.73 m^2^ in favour of the SGLT2i group (*p* = 0.046).

Albuminuria tended to be lower in the SGLT2i group, but the change over time in UACR was not significantly different between groups (*p* = 0.52) as was the rate of albuminuria category improvement (HR 1.07; 95% CI 0.88–1.31).

## 4 DISCUSSION

This large, retrospective multicenter study of patients with T2D and CKD provides real‐world evidence that initiation of SGLT2i is associated with a significantly slower decline in kidney function compared with other glucose‐lowering medications, including GLP‐1RA. SGLT2i use led to an attenuation in the rate of eGFR decline of about 1 mL/min/1.73 m^2^/year and a significantly lower incidence of adverse kidney outcomes, including a ≥40% or ≥57% reduction in eGFR. Albuminuria also improved more in the SGLT2i group, with significant reductions in UACR and greater likelihood of UACR class improvement.

These findings reinforce the kidney‐protective effects of SGLT2i in clinical practice. Better kidney outcomes were observed despite a slightly smaller reduction in HbA1c in the SGLT2i group, suggesting that renoprotection is independent of glycemic control and may be partially driven by haemodynamic effects. Comparisons with GLP‐1RA showed that SGLT2i conferred superior preservation of eGFR, with a mean annual slope difference of 0.62 mL/min/1.73 m^2^, supporting the preferential use of SGLT2i in patients with CKD when both classes are being considered, as suggested by the 2025 ADA standards of care.^21^

A major renoprotective mechanism of SGLT2i is the restoration of tubuloglomerular feedback via reduced sodium reabsorption in the proximal tubule, leading to afferent arteriole vasoconstriction and a reduction in intraglomerular pressure. Other main glycemia‐independent effects include reduced tubular workload and hypoxia, anti‐inflammatory and antifibrotic effects, as well as ketogenesis and metabolic reprogramming.^22^

These real‐world results are in line with findings from large randomized trials such as CREDENCE and the diabetic subgroups of DAPA‐CKD and EMPA‐KIDNEY,^23^, ^24^, ^25^ but uniquely extend the evidence base to routine clinical settings and to a broader population. The inclusion criteria in this observational analysis were less restrictive, allowing enrolment of patients across a wider age range, with a different spectrum of CKD, such as those with moderately reduced eGFR and/or elevated albuminuria. While dedicated trials enrolled patients with more advanced CKD, a large proportion of the patients in the present study had CKD stage G2A2/3. Indeed, CKD was defined more often by a raised albuminuria with normal eGFR in this real‐world study and most patients with reduced eGFR were normoalbuminuric. Moreover, patients frequently had multiple comorbidities, concomitant use of various GLM, and uncontrolled HbA1c at baseline, reflecting the complexity of diabetes care in everyday clinical practice. These differences enhance the generalizability of the findings and provide reassurance that the renal benefits of SGLT2i extend beyond the controlled trial environment to a broader population typically seen in routine care.

Our data are consistent with prior real‐world studies assessing kidney outcomes in people with T2D and CKD who initiated an SGLT2i or other diabetes drugs in different countries and healthcare systems.^26^, ^27^, ^28^, ^29^, ^30^ Compared with the available literature, our study is the largest analysing outcomes based on both eGFR and UACR in people with T2D selected for having CKD at baseline. A large study from Taiwan^31^ showed SGLT2i to be associated with a stronger reduction in CKD progression among patients with macroalbuminuria compared with those without, but the same finding has not been replicated by others.^32^ While the benefits of SGLT2i on CKD progression were well documented in more advanced stages (eGFR < 60 mL/min/1.73 m^2^), the relative novelty, here, is that the slowing of eGFR decline can be achieved in the early stages of kidney impairment, even before substantial eGFR reductions. An early identification of microalbuminuria, once considered a mild wake‐up call, becomes a concrete therapeutic opportunity; starting an SGLT2i early can block the natural trajectory of CKD and support a paradigm shift in the management of diabetes with subclinical organ damage.

The study strengths rely on the robust methodological approach to biases inherent to observational research. In real‐world datasets, treatment allocation is not randomized and is influenced by multiple clinical and demographic factors, leading to confounding by indication. This was evident from the baseline differences between the two cohorts before PSM: patients initiated on SGLT2i had a more severe disease state, but also a better kidney status, due to the fact that use of SGLT2i in patients with reduced eGFR was contraindicated for part of the data collection period. PSM was essential to create well‐balanced cohorts with similar baseline characteristics, mimicking the comparability of groups typically achieved in RCTs. Furthermore, real‐world data often contain missing values for key clinical variables, which can bias results or reduce statistical power if cases with incomplete data are excluded. MICE allowed the creation of 10 imputed datasets, which were each analysed and then pooled, thereby preserving the variability and uncertainty associated with missingness and ensuring the integrity of the analyses. The combination of PSM and MICE minimized confounding and information bias, supporting the internal validity of the findings and allowing for reliable estimation of the effect of SGLT2i on kidney outcomes in a non‐randomized, real‐world context.

However, residual confounding cannot be excluded, and the absence of randomization limits causal inference. Furthermore, the lack of data on medication adherence and potential ascertainment bias in follow‐up eGFR and UACR measurements may affect outcome precision. It should be noted that, while all eGFR‐based secondary outcomes are consistent with the primary endpoint, eGFR‐independent secondary outcomes (e.g., albuminuria‐based ones) are only nominally significant because no adjustment of type I error inflation due to multiple testing was performed, and should therefore be considered exploratory.

Another limitation of this study is the lack of information on cardiovascular events and vital status, precluding an analysis of competing outcomes, which are particularly relevant in a population with T2D and CKD, where the risk of cardiovascular death often exceeds the risk of progression to kidney failure.^33^, ^34^ In the context of kidney outcome analyses, failing to account for competing risks may lead to overestimation of the incidence of renal events. While the study provides strong evidence for the nephroprotective effects of SGLT2i, the absence of data on these competing outcomes limits the ability to assess the full clinical trajectory of patients and precludes the evaluation of comprehensive cardiorenal endpoints, which are increasingly recognized as clinically meaningful. Yet, under the assumption that treatment with SGLT2i does not worsen cardiovascular outcomes and survival, our estimates of renal protection by SGLT2i is conservative.

The lack of information on adverse events during treatment is another limitation of the present study, because it was impossible to perform an analysis of the risk/benefit balance. While the safety profile of SGLT2i is extensively known from trials and post‐marketing surveillance, other safety considerations in the comparator group (e.g., hypoglycemia and gastrointestinal side effects) may be of interest.

Head‐to‐head comparisons between SGLT2i and GLP‐1RA with kidney outcomes as primary endpoints are timely and relevant in the current diabetes and nephrology care. Both drug classes are now recommended for patients with T2D and high cardiorenal risk, yet direct comparative evidence is limited. Some real‐world studies have reported better kidney outcomes in new users of SGLT2i versus GLP‐1RA for the care of T2D,^35^, ^36^ including a prior analysis from the DARWIN‐Renal database.^18^ While large‐scale cardiovascular outcome trials and dedicated kidney trials have robustly demonstrated the renoprotective effects of SGLT2i, the evidence for GLP‐1RA is less definitive, often emerging from secondary trial analyses^37^ or metanalyses.^38^ The FLOW study demonstrated that semaglutide significantly reduced the risk of kidney disease progression by 24% and major cardiovascular events by 18% in patients with T2D and albuminuric CKD,^13^ thereby providing the first robust, randomized evidence of a GLP‐1RA conferring kidney protection in this population. In light of these findings, the comparison of SGLT2i versus GLP‐1RA becomes even more clinically pertinent. While FLOW was a rigorous placebo‐controlled randomized trial with strict inclusion criteria and protocol‐driven follow‐up, our observational study reflects everyday clinical practice, encompassing a different population with heterogeneity in comorbidities, background therapies, and disease severity. Despite the smaller sample size for the head‐to‐head comparison, the observed benefit of SGLT2i on eGFR slope in this study supports the notion that SGLT2i may offer more pronounced or faster‐acting nephroprotection, particularly in patients with established CKD. The median follow‐up of 1.6 years is relatively long in the current real‐world scenario but may be too short for some drugs to show their maximum protective potential, especially for outcomes such as ESKD.

In a prior analysis of the DARWIN‐Renal database, GLP‐1RA reduced the rates of new‐onset macroalbuminuria in participants without CKD at baseline compared with SGLT2i.^18^ Here, in the presence of baseline CKD, we found no difference in UACR change between new‐users of SGLT2i or GLP‐1RA, despite semaglutide in the FLOW trial significantly reduced albuminuria.^13^ This may be attributable to several factors, including the reduced statistical power in our analysis, the very small proportion of patients on semaglutide, the different population compared with the FLOW trial (where all participants had to have albuminuria at baseline), and the variability of UACR measure in real‐life conditions.

These considerations reinforce the need for head‐to‐head trials or well‐powered real‐world comparative effectiveness studies with sufficient follow‐up to guide optimal sequencing or combination strategies, especially in patients with both high cardiovascular and renal risk. In this context, the current study's exploratory analysis represents a timely and clinically meaningful contribution to ongoing decision‐making in the post‐FLOW landscape.

In conclusion, this study provides compelling real‐world evidence that SGLT2i, better than active comparators and GLP‐1RA, significantly attenuate the progression of kidney disease in patients with T2D and CKD, reinforcing their role as a foundational therapy in this high‐risk population.

## AUTHOR CONTRIBUTIONS

GPF conceptualized the study, interpreted the analyses, visualized the results, searched the literature and wrote the manuscript. EL conceptualized the study, performed and interpreted the analyses, visualized the results, developed the methodological pipeline, searched the literature and contributed to writing the manuscript. FB, GA, GTR, RA and MG collected and researched data, contributed to data interpretation discussion and revised the manuscript. MLM, AA and AS conceptualized the study, coordinated and supervised data collection, acquired funding for the analysis, and critically reviewed the manuscript for important intellectual content. All authors contributed intellectually to this study and critically revised the scientific content of the manuscript. All authors had access to all the data of the study, approved the final manuscript as submitted, agreed to be accountable for all aspects of the work and had final responsibility for the decision to submit for publication. GPF and EL are the guarantors of this work and, as such, had full access to all the data in the study, verified the data and took responsibility for the integrity of the data and the accuracy of the data analysis.

## FUNDING INFORMATION

This study was promoted by the Italian Diabetes Society and partly supported by AstraZeneca. The external funding source had no role in study design and conduction, data collection, analysis and interpretation, manuscript writing and decision to publish.

## CONFLICT OF INTEREST STATEMENT

GPF received fees for lectures, consultancy, or advisory board from AstraZeneca, Boehringer, Lilly, Guidotti, Novartis, Novo Nordisk, Sanofi. MLM received fees for lectures, consultancy or advisory board from Amgen, AstraZeneca, Boehringer Ingelheim, Daiichi Sankyo, Eli Lilly, Guidotti, Merck Sharp & Dohme, Novartis, Novo Nordisk, Sanofi and Servier. GTR is on the advisory board and does consultancy and lectures for Novo Nordisk, AstraZeneca, Sanofi, Boehringer, Lilly, Mundipharma and Sanchio. FB received lecture or consultancy fees from Boehringer‐Ingelheim, GSK, Lilly and Novo Nordisk. AA received research grants, lecture, or advisory board fees from Merck Sharp & Dome, AstraZeneca, Novartis, Boeringher‐Ingelheim, Sanofi, Mediolanum, Janssen, Novo Nordisk, Lilly, Servier and Takeda. AS served on the advisory board of Novo Nordisk, Sankyo, and Sanofi and received speaker fees from Bayer, Lilly, Novo Nordisk, and Sanofi. EL, MG, GA RA has nothing to disclose.

## Supporting information


**Data S1.** Supporting information.

## Data Availability

Restrictions apply to the availability of crude data used for this study. Aggregated data are available upon reasonable request via email to the corresponding author.
