# Supplementary material for: Long‐term preservation of kidney function with SGLT‐2 inhibitors versus comparator drugs in people with type 2 diabetes and chronic kidney disease
Source: Diabetes Obes Metab. 2025 Jul 2;27(9):5182–91. doi: 10.1111/dom.16569 (PMC12326893; doi:10.1111/dom.16569)
Supplement: Supplementary file 1 — Data S1. Supporting information. [file DOM-27-5182-s001.docx]

**Appendix**

**DARWIN-Renal study Investigators**

ABRUZZO. Maria Pompea Antonia Baldassarre, Gloria Formoso, Agostino Consoli (Endocrinology, Diabetes and Metabolism, University Chieti-Pescara). Sara Morgante, Antonella Zugaro, Marco Giorgio Baroni (Diabetes and Andrology Unit, San Salvatore Hospital, University of L'Aquila).

CALABRIA. Francesco Andreozzi (Department of Medical and Surgical Sciences, University Magna Graecia of Catanzaro, Catanzaro).

CAMPANIA. Adriano Gatti (Diabetology Service, ASL Napoli1). Stefano De Riu (Diabetology Unit ASL Napol1 Centro). Andrea Del Buono (Diabetology Unit, ASL Caserta).

EMILIA ROMAGNA. Raffaella Aldigeri, Riccardo Bonadonna (Division of Endocrinology and Metabolic Diseases, Azienda Ospedaliera-Universitaria di Parma, Department of Medicine and Surgery, University of Parma), Alessandra Dei Cas (Division of Nutritional and Metabolic Sciences, Azienda Ospedaliero-Universitaria di Parma, University of Parma), Angela Vazzana, Monica Antonini, Valentina Moretti (Division of Endocrinology and Metabolic Diseases, Azienda Ospedaliera-Universitaria di Parma).

FRIULI VENEZIA GIULIA. Patrizia Li Volsi (Section of Endocrinology and Metabolism, Azienda Sanitaria Friuli Occidentale). Miranda Cesare, Giorgio Zanette (Section of Endocrinology and Metabolism, Pordenone Hospital, Azienda Sanitaria Friuli Orientale).

LAZIO. Silvia Carletti, Paola D'Angelo (Diabetology Unit, Sandro Pertini Hospital, ASL Roma2). Gaetano Leto, Frida Leonetti (Diabetology Unit Latina, Department of Medical-Surgical Sciences and Biotechnologies, Sapienza University, Rome, Italy). Luca D'Onofrio, Ernesto Maddaloni, Raffaella Buzzetti (Diabetology Unit, University of Rome "La Sapienza"). Simona Frontoni (Unit of Endocrinology, Diabetes and Metabolism, S. Giovanni Calibita Fatebenefratelli Hospital, Department of Systems Medicine, University of Rome Tor Vergata, Rome, Italy). Maria Gisella Cavallo, Ilaria Barchetta (Diabetology Unit, Department of Experimental Medicine, Sapienza University, Rome, Italy). Susanna Morano, Tiziana Filardi (University of Rome "La Sapienza", Dept. of Experimental Medicine, Unit of Diabetes and Complications, Clinica Medica V, Azienda Policlinico Umberto I, Roma). Umberto Capece, Andrea Giaccari (Endocrinology and Diabetology, IRCCS Agostino Gemelli University Hospital Foundation, Department of Translational Medicine and Surgery, Catholic University of the Sacred Heart, Rome).

LOMBARDIA. Antonio C. Bossi (Unit of Diabetology, Humanitas Gavazzeni Institute, Bergamo). Giancarla Meregalli (Unit of Diabetology and Metabolic Diseases, Azienda Socio-Sanitaria Territoriale Bergamo Ovest, Treviglio, Bergamo). Fabrizio Querci (Diabetology Unit, Alzano Lombardo Hospital). Alessia Gaglio, Veronica Resi, Emanuela Orsi (Diabetes Unit, Foundation IRCCS Cà Granda Ospedale Maggiore Policlinico, Milan). Stefano Fazion (Diabetology and Metabolic Disease, ASST Mantova). Ivano G. Franzetti (Endocrinology and Diabetology Unit ASST Valle Olona). Cesare Berra (Diabetology and Endocrinology Unit, IRCCS Multimedica, Milan).

MARCHE. Silvia Manfrini (Diabetology Unit, Senigallia). Gabriella Garrapa, Giulio Lucarelli, Lara Riccialdelli (Diabetology Unit, Fano). Elena Tortato (Diabetology and Metabolic Disease, INRCA Ancona).

PIEMONTE. Marco Zavattaro, Gianluca Aimaretti (Division of Endocrinology, Maggiore della Carità Hospital, Department of Translational Medicine, University of Piemonte Orientale, Novara). Franco Cavalot (Diabetes and Metabolic Diseases Unit, San Luigi Gonzaga University Hospital, Turin). Guglielmo Beccuti, Fabio Broglio (Unit of Diabetology and Metabolism, Department of Medical Sciences, University of Turin, 10123 Torino, Italy.).

TRENTINO-ALTO ADIGE. Bruno Fattor (Diabetology Service, Bolzano Hospital).

PUGLIA. Giuliana Cazzetta (Diabetology Unit Tricase (Lecce)). Olga Lamacchia (Department of Medical and Surgical Sciences, University of Foggia). Anna Rauseo, Salvatore De Cosmo (Fondazione IRCCS Casa Sollievo della Sofferenza, San Giovanni Rotondo, Italy).

SARDEGNA. Rosella Cau, Mariangela Ghiani (UO diabetologia Quartu SE, ASL 8 Cagliari).

SICILIA. Antonino Di Benedetto (Diabetology Unit, University Hospital G. Martino, Messina). Antonino Di Pino, Salvatore Piro, Francesco Purrello (Internal Medicine, Garibaldi Nesima Hospital, Department of Clinical and Experimental Medicine, University of Catania). Lucia Frittitta, Agostino Milluzzo (Center for Diabetes and Obesity, Garibaldi Nesima Hospital, Department of Clinical and Experimental Medicine, University of Catania). Giuseppina Russo (Metabolic Disease and Internal Medicine, University Hospital of Messina).

TOSCANA. Anna Solini (Department of Surgical, Medical, Molecular and Critical Area Pathology, University of Pisa). Monia Garofolo, Giuseppe Penno, Stefano Del Prato (Department of Clinical and Experimental Medicine, University of Pisa, Pisa). Roberto Anichini (Diabetes Unit, Area Pistoiese USL Toscana Centro).

VENETO. Gian Paolo Fadini, Angelo Avogaro (Department of Medicine, University of Padova). Lucia Gottardo (Unit of Hypertension and Endocrine-Metabolic-Angiologic Disease, AULSS3 Venice). Mauro Rigato, Agostino Paccagnella (Diabetology Units, Conegliano and Treviso). Marco Strazzabosco (Endocrine, Metabolic and Nutrition Disease, Vicenza Hospital). Massimo Cigolini, Enzo Bonora (Division of Endocrinology, Diabetes and Metabolic Diseases, Department of Medicine, University of Verona).

**Table S1. Intermediate outcomes**. The mean difference is presented for the SGLT2i versus comparator groups (negative values imply a reduction in the SGLT2i group).

| **Outcome** | **Mean difference (95% C.I.)** | **p-value** |
| --- | --- | --- |
| HbA1c, % | 0.16 (0.10; 0.23) | <0.001 |
| Body weight, kg | -0.72 (-1.87; 0.42) | 0.216 |
| Systolic blood pressure, mm Hg | -1.65 (-2.72; -0.58) | 0.003 |
| Diastolic blood pressure, mm Hg | -0.35 (-0.92; 0.21) | 0.223 |

**Table S2. Characteristics of new-users of SGLT-2 inhibitors or GLP-1RA**. Data are shown before and after propensity score matching (PSM). The standardized mean difference (SMD) is reported to evaluate the balance between groups.

|  | **Before PSM** | | | **After PSM** | | |
| --- | --- | --- | --- | --- | --- | --- |
|  | **SGLT2i** | **GLP-1RA** | **SMD** | **SGLT2i** | **GLP-1RA** | **SMD** |
| **Number** | 3014 | 1632 |  | 1233 | 1233 |  |
| **Demographics** |  |  |  |  |  |  |
| Sex male, % | 2007 (66.6) | 1044 (64.0) | 0.06 | 812 (65.9) | 808 (65.5) | <0.01 |
| Age, years | 62.9 (8.7) | 63.9 (8.8) | 0.11 | 63.1 (8.9) | 63.4 (9.2) | 0.03 |
| Diabetes duration, years | 13.2 (9.0) | 12.1 (8.6) | 0.13 | 11.3 (8.4) | 11.7 (8.4) | 0.04 |
| **Anthropometrics** |  |  |  |  |  |  |
| Weight, kg | 91.4 (18.6) | 95.4 (18.5) | 0.21 | 93.0 (19.0) | 93.1 (17.5) | <0.01 |
| Body mass index, kg/m2 | 32.5 (5.9) | 33.9 (6.0) | 0.23 | 32.9 (6.0) | 33.0 (5.5) | 0.02 |
| **Risk factors and laboratory** |  |  |  |  |  |  |
| Systolic blood pressure, mm Hg | 140.4 (19.8) | 140.7 (19.5) | 0.01 | 140.7 (20.4) | 140.5 (19.2) | 0.01 |
| Diastolic blood pressure, mm Hg | 79.4 (10.5) | 80.2 (10.3) | 0.08 | 80.1 (10.7) | 80.1 (10.3) | <0.01 |
| Fasting plasma glucose, mg/dl | 177.6 (61.0) | 165.8 (49.8) | 0.21 | 168.4 (54.1) | 167.9 (50.3) | 0.01 |
| HbA1c, % | 8.5 (1.5) | 8.0 (1.3) | 0.33 | 8.1 (1.4) | 8.1 (1.3) | <0.01 |
| Total cholesterol, mg/dl | 172.1 (43.2) | 170.5 (43.1) | 0.04 | 171.7 (40.9) | 171.0 (43.0) | 0.02 |
| HDL cholesterol, mg/dl | 44.5 (12.5) | 44.5 (12.9) | <0.01 | 44.6 (11.9) | 44.9 (13.0) | 0.02 |
| LDL cholesterol, mg/dl | 92.1 (34.8) | 91.0 (34.8) | 0.03 | 92.4 (34.6) | 91.6 (34.6) | 0.02 |
| Triglycerides, mg/dl | 181.9 (125.3) | 181.2 (116.4) | <0.01 | 179.9 (117.4) | 178.4 (117.6) | 0.01 |
| eGFR (ml/min/1.73 m^2^) | 78.2 (21.6) | 68.1 (24.2) | 0.45 | 74.7 (21.3) | 73.9 (23.4) | 0.04 |
| Albumin excretion rate, mg/g | 252.2 (628.7) | 234.5 (630.9) | 0.03 | 226.7 (587.0) | 235.0 (598.8) | 0.01 |
| eGFR slope (ml/min/1.73 m^2^/year) | -1.4 (15.2) | -2.5 (11.9) | 0.08 | -2.1 (16.1) | -2.0 (11.8) | <0.01 |
| **Complications** |  |  |  |  |  |  |
| eGFR >15 and < 60 ml/min/1.73 m^2^ | 877 (29.1) | 815 (49.9) | 0.44 | 459 (37.2) | 473 (38.4) | 0.02 |
| Pathologic albuminuria, % | 2354 (78.1) | 1016 (62.3) | 0.36 | 883 (71.6) | 869 (70.5) | 0.03 |
| Diabetic Retinopathy; % | 829 (27.5) | 345 (21.1) | 0.15 | 245 (19.9) | 255 (20.7) | 0.02 |
| Diabetic macular edema, % | 137 (4.5) | 47 (2.9) | 0.09 | 35 (2.8) | 40 (3.2) | 0.02 |
| Stroke / TIA; % | 55 (1.8) | 36 (2.2) | 0.03 | 17 (1.4) | 24 (1.9) | 0.04 |
| Carotid atherosclerosis, % | 774 (25.7) | 416 (25.5) | <0.01 | 290 (23.5) | 311 (25.2) | 0.04 |
| Ischemic heart disease; % | 549 (18.2) | 241 (14.8) | 0.09 | 197 (16.0) | 192 (15.6) | 0.01 |
| Left ventricular hypertrophy, % | 328 (10.9) | 165 (10.1) | 0.03 | 128 (10.4) | 127 (10.3) | <0.01 |
| Heart failure, % | 141 (4.7) | 65 (4.0) | 0.03 | 57 (4.6) | 56 (4.5) | <0.01 |
| Any site revascolarization, % | 373 (12.4) | 179 (11.0) | 0.04 | 148 (12.0) | 135 (10.9) | 0.03 |
| Microvascular complications, % | 3014 (100.0) | 1632 (100.0) | <0.01 | 1233 (100.0) | 1233 (100.0) | <0.01 |
| Macrovascular complications, % | 1338 (44.4) | 657 (40.3) | 0.08 | 515 (41.8) | 502 (40.7) | 0.02 |
| Established CVD, % | 651 (21.6) | 301 (18.4) | 0.08 | 244 (19.8) | 233 (18.9) | 0.02 |
| **Glucose lowering medications** |  |  |  |  |  |  |
| Metformin, % | 2260 (75.0) | 1213 (74.3) | 0.02 | 990 (80.3) | 986 (80.0) | <0.01 |
| Sulphonylurea / repaglinide, % | 170 (5.6) | 226 (13.8) | 0.30 | 129 (10.5) | 139 (11.3) | 0.03 |
| Pioglitazone, % | 39 (1.3) | 54 (3.3) | 0.14 | 28 (2.3) | 28 (2.3) | <0.01 |
| Acarbose, % | 18 (0.6) | 7 (0.4) | 0.02 | 5 (0.4) | 6 (0.5) | 0.01 |
| Bolus insulin, % | 1153 (38.3) | 91 (5.6) | 0.79 | 95 (7.7) | 91 (7.4) | 0.01 |
| Basal insulin, % | 1579 (52.4) | 529 (32.4) | 0.41 | 377 (30.6) | 378 (30.7) | <0.01 |
| **Other medications** |  |  |  |  |  |  |
| Statins, % | 1928 (64.0) | 1031 (63.2) | 0.02 | 779 (63.2) | 767 (62.2) | 0.02 |
| Anti-platelet agents, % | 1492 (49.5) | 758 (46.4) | 0.06 | 575 (46.6) | 553 (44.8) | 0.04 |
| RAS blockers, % | 2178 (72.3) | 1148 (70.3) | 0.04 | 879 (71.3) | 871 (70.6) | 0.01 |
| Beta blockers, % | 1083 (35.9) | 594 (36.4) | <0.01 | 433 (35.1) | 429 (34.8) | <0.01 |
| Calcium channel inhibitors, % | 875 (29.0) | 492 (30.1) | 0.02 | 353 (28.6) | 369 (29.9) | 0.03 |
| Diuretics, % | 1131 (37.5) | 683 (41.9) | 0.09 | 480 (38.9) | 474 (38.4) | <0.01 |
| Anticoagulants, % | 106 (3.5) | 71 (4.4) | 0.04 | 45 (3.6) | 47 (3.8) | <0.01 |

**Figure S1. Change in eGFR over time before and after index date**.

**Figure S2. eGFR slopes before and after index date.** The graphs shows the comparison between new-users of SGLT-2 inhibitors (SGLT2i) and GLP-1RA.
